# Cross-sectional assessment of mild cognitive impairment in pre-dialysis chronic kidney disease and its association with inflammation and changes seen on MRI: what the eyes cannot see

**DOI:** 10.1590/2175-8239-JBN-2021-0194

**Published:** 2022-02-14

**Authors:** Leopoldo Antônio Pires, Ana Laura Maciel de Almeida, Marilise de Andrade Paraízo, José Otávio do Amaral Corrêa, Débora dos Santos Dias, Neimar da Silva Fernandes, Danielle Guedes Andrade Ezequiel, Rogério Baumgratz de Paula, Natália Maria da Silva Fernandes

**Affiliations:** 1 Universidade Federal de Juiz de Fora, Empresa Brasileira de Serviços Hospitalares, Juiz de Fora, MG, Brasil.; 2 Universidade Federal de Juiz de Fora, Núcleo Interdisciplinar de Estudos, Pesquisas em Nefrologia (NIEPEN), Juiz de Fora, MG, Brasil.

**Keywords:** Cognitive Dysfunction, Renal Insufficiency, Chronic, Inflammation, Magnetic Resonance Imaging, Disfunção Cognitiva, Insuficiência Renal Crônica, Inflamação, Imageamento por Ressonância Magnética

## Abstract

**Introduction:**

Mild cognitive impairment (MCI) is a prevalent and underdiagnosed condition in chronic kidney disease (CKD), that shares common pathophysiological factors such as chronic inflammation.

**Objective:**

To evaluate the association of MCI in CKD stages 1-5 using inflammatory markers and changes by magnetic resonance imaging (MRI).

**Patients and Methods:**

Cross-sectional study in adult patients with pre-dialysis CKD. MCI was assessed by the Montreal Cognitive Assessment (MoCA) and the estimated glomerular filtration rate (eGFR) by the Chronic Kidney Disease Epidemiology Collaboration equation. Sociodemographic and clinical data were collected from medical records. The cytokines IL-4, IL-6, IL-17, TNF-α and hs-CRP were determined. Brain MRI was performed in a 1.5 Tesla device, without paramagnetic contrast. A descriptive analysis followed by a comparison of abnormal versus normal MoCA scores among all studied variables. A linear regression analysis was performed using MoCA as a dependent variable, adjusted for confounding factors.

**Results:**

Of 111 invited patients, eighty completed the neuropsychological assessment and 56 underwent MRI, and were included in the study. Mean age was 56.3 ± 8.3 years and 51.8% (n = 29) had altered MoCA. When compared to the group with normal MoCA, the group with altered MoCA had higher levels of IL-6 and IL-17. There was no correlation between altered MoCA with eGFR or with MRI abnormalities.

**Conclusão:**

MCI assessed by MoCA was prevalent in patients with pre-dialysis CKD, it was associated with inflammation and showed no correlation with MRI changes.

## Resumo

**Introdução:**

O comprometimento cognitivo leve (CCL) é prevalente e subdiagnosticado na doença renal crônica (DRC), condição com a qual compartilha fatores fisiopatológicos como a inflamação crônica.

**Objetivo:**

Avaliar a associação do CCL na DRC estágios 1 a 5, com marcadores inflamatórios e alterações de exames de imagem por ressonância magnética (RM).

**Pacientes e métodos:**

Estudo transversal em pacientes adultos, com DRC pré-dialítica. CCL foi avaliado pelo *Montreal Cognitive Assessment* (MoCA) e a taxa de filtração glomerular estimada (TFGe), pela equação do CKD-EPI. Dados sociodemográficos e clínicos foram coletados nos prontuários médicos. Dosadas citocinas IL-4, IL-6, IL-17, o TNF-α e PCR-us. A RM do encéfalo foi realizada em aparelho de 1,5 Tesla, sem contraste. Realizada análise descritiva seguida por comparação de pontuações do MoCA anormais versus normais entre todas as variáveis estudadas. A regressão linear foi realizada usando MoCA como uma variável dependente, ajustada para fatores de confusão.

**Resultados:**

De 111 pacientes convidados, oitenta completaram a avaliação neuropsicológica, 56 realizaram RM, tendo sido incluídos no estudo. A média de idade foi de 56,3 ± 8,3 anos e 51,8% (n = 29) apresentavam MoCA alterado. Quando comparado ao grupo MoCA normal, o grupo MoCA alterado apresentou níveis mais elevados de IL-6 e IL-17. Não houve correlação entre MoCA alterado com TFGe nem com anormalidades na RM. Nos modelos ajustados, a IL-6 foi preditor independente do MoCA alterado

**Conclusão:**

O CCL avaliado pelo MoCA foi prevalente em pacientes com DRC pré-dialítica, associou-se com inflamação e não apresentou correlação com alterações da RM.

## Introduction

One of humanity’s achievements was the improvement in the diagnosis and treatment of maternal, child and infectious diseases, significantly reducing morbidity and mortality rates. This phenomenon led to the predominance of non-communicable chronic diseases (NCDs), highlighting systemic arterial hypertension (SAH) and diabetes mellitus (DM), a favorable scenario for the increased prevalence of cardiovascular diseases (CVD), chronic kidney disease (CKD) and dementia syndromes^1^.

The prevalence of CKD has increased significantly, being estimated at around 11% of the world population^2^. A similar phenomenon occurs with cognitive impairment (CI) and dementias, particularly in low and middle-income countries, in which CI is associated with population aging, low socioeconomic and educational levels and a higher frequency of potentially risk factors modifiable, such as SAH, sedentary lifestyle, alcohol abuse, etc^3,4^.

In Latin America, dementias are a growing problem, as a result of the high levels of illiteracy and the demographic transition observed in that continent^5^. Among the dementias, Alzheimer’s disease accounts for about two thirds of the cases, followed by vascular dementia, mixed dementia, Lewy body dementia and frontotemporal degeneration^6,7^. As a general rule, we use specific protocols to assess CI. In these, the most cited cognitive screening instruments are the Mini Mental State Examination (MMSE)^8^ and the Montreal Cognitive Assessment (MoCA)^9^, associated with the assessment of functional activities, usually performed using the functional activities questionnaire by Pfeffer et al.^10^, in addition to laboratory tests for syphilis (VDRL), vitamin B12 dosing, thyroid hormones and human immunodeficiency virus (HIV) serology. These are associated with running neuroimaging exams, represented by computerized tomography (CT) and/or magnetic resonance (MR) of the brain, aiming to exclude reversible causes of CI^11^.

In recent years, the association of CKD categories 4 and 5 with dementia has been reported by different authors^12-14^. Preceding the diagnosis of dementia, initially mild and progressive CI occurs, often undiagnosed. Mild cognitive impairment (MCI), described by Petersen et al.^15^, is defined as cognitive impairment without functional repercussions, being an intermediate condition between normality and dementia. Since then, there have been several reports of CI at different stages of CKD, affecting up to two-thirds of this population. Etiologically, MCI is attributed to factors such as uremic toxins, inflammation and oxidative stress, among others^16,17^. These factors determine endothelial dysfunction and, later, vascular lesions identifiable by brain MRI and characterized by white matter hyperintensities, subclinical cerebral infarcts or cortical atrophies^18,19^. Therefore, in initial cases of CI in CKD, these changes, of a functional nature, cannot be identified by MRI, making early diagnosis difficult.

We know that CI impacts the treatment of CKD, as it interferes with the understanding of the guidelines from healthcare professionals, reducing adherence to dietary guidelines and pharmacological treatment in this population. Thus, it is essential to diagnose MCI as early as possible^17,19,20^. The hypothesis is that, in CKD, cognitive alterations are initially of a functional nature and, therefore, not identifiable by MRI. Given the above, this study focuses on evaluating the MCI detected by MoCA, inflammatory markers and changes in brain MRI, in patients with CKD under conservative treatment.

## Patients and Methods

This study was approved by the Research Ethics Committee (CEP) of the Federal University of Juiz de Fora, under protocol nº. 183,387.

This is a cross-sectional study of a database created for cognitive analysis of patients with pre-dialysis CKD, called NEFROCOG. For this base, we invited the patients scheduled for clinical consultations at the Hiperdia Minas Center in Juiz de Fora-MG (CHM-JF) from March 2013 to December 2014. The patients were contacted by telephone and scheduled for individual neuropsychological evaluation, on a day different from that of their clinical appointment.

We used a convenience sample, consisting of 111 patients seen by the CHM-JF of secondary care in SAH, DM and CKD. Of these, 14 were participated in a pilot project - carried out to calibrate the tests. The following were defined as inclusion criteria: patients with CKD in categories 1 to 5, undergoing conservative treatment (non-dialysis); age > 21 and < 65 years; they all agreed to participate in the study and signed the Informed Consent Form (ICF). We excluded those patients with a previous history of cerebrovascular accident (CVA); infectious or degenerative diseases of the Central Nervous System (CNS); presence of delirium and/or psychotic disorders; previous history of traumatic brain injury; visual and auditory disorders that prevented the tests from being carried out; HIV and/or Acquired Immunodeficiency Syndrome (AIDS); and contraindication or intolerance to performing MRI.

The sociodemographic and clinical variables analyzed were collected from the patients’ records or investigated with them. The following variables were evaluated: age, hemispheric dominance, educational level (< 4 years or > 4 years), sex, color, marital status and family income. Clinical variables included: SAH, DM, CVD, hypothyroidism, depression, smoking, alcoholism, sedentary lifestyle, CKD category and use of medications, namely: opiates, anti-Parkinsonians, hypnotics, antiepileptics, benzodiazepines, antidepressants, angiotensin converting enzyme inhibitors (ACEI), angiotensin receptor blockers (ARB), beta blockers, calcium channel blockers, adrenergic blockers, vasodilators, diuretics, statins, fibrate, vitamin D, oral antidiabetics, insulin, erythropoietin, iron, levothyroxine, nitrates and aspirin.

For laboratory variables, we collected blood samples by venipuncture after the patient fasted for 12 hours during the same period of the clinical evaluation. We evaluated the following: hemoglobin, urea, creatinine, sodium, potassium, blood glucose, glycated hemoglobin, calcium, phosphorus, magnesium, alanine aminotransferase/aspartate aminotransferase (ALT/AST), total cholesterol, high-density lipoproteins (HDL), low-density lipoproteins (LDL), triglycerides, albumin, uric acid, ferritin, transferrin saturation index (IST), parathormone (PTH), thyroid stimulating hormone (TSH), vitamin B12, folic acid, vitamin D3, ultrasensitive C-reactive protein (US-CRP), VDRL, fluorescent treponemal antibodies (FTA-ABS), hepatitis B surface antigen (HBsAg R), antibody to hepatitis B surface antigen (Anti-HBs), antibody to hepatitis C surface antigen (anti -HCV), estimated glomerular filtration rate (eGFR) and proteinuria.

Inflammatory markers represented by interleukins (IL) IL-4, IL-6 and IL-17 and by tumor necrosis factor alpha (TNF-α) were also evaluated. For this purpose, 10 mL blood samples were collected in tubes without anticoagulant. The tubes were centrifuged at 3,500 rpm and the collected sera were frozen at-86°C. The measurements of these markers were performed using the Enzyme Linked Immune Sorbent Assay (ELISA) technique. The samples were quantified by comparison with recombinant standard curves (antibody and recombinant concentrations as per manufacturer’s recommendations, PeProtech Inc, New Jersey).

### Evaluation and study instruments

Two medical professionals (one neurologist and on psychologist) performed the assessments, conducted in a silent environment, in a single session of approximately one hour.

We ran a structured anamnesis and applied clinical depression questionnaires for the tests were applied in the same sequence for all patients and corrected only by the neurologist/psychologist examiner. The sequence of the neuropsychological tests’ application considered both the mental wear of the individual when performing each test and the interference of one test with another, which could generate, for example, learning. We must also consider that some tests are distracting.

The Mini International Neuropsychiatric Interview/Mini International Neuropsychiatric Interview-Plus (M.I.N.I/M.I.N.I-PLUS)^21^ tests were used to diagnose depression and MoCA, in the Brazilian version^22^, to diagnose CI. To interpret the MoCA, the cutoff point adopted for the Brazilian population (≤ 25 points) was reduced to ≤ 24 points, as a strategy to adjust the sample’s educational level. The choice of MoCA was due to the good sensitivity of the test to detect MCI when compared to the MMSE^17^.

The Pfeffer et al.^10^ questionnaire was also used to assess the patient’s functionality. This test was applied to the companion simultaneously, with the patient’s assessment or over the telephone.

The questionnaires and tests, the functions and domains assessed, as well as the cutoff point used for their correction, are shown in Chart 1. The outcome assessed was CI assessed by the MoCA.

**Chart 1:** Chart showing the tests and questionnaires used in the study

Tests/Questionnaires	Description/Functions	Score
Montreal Cognitive Assessment (MoCA)* Nasreddine et al.^9^	Global cognitive assessment: visuo-spatial skills, executive functions, language, memory, attention/orientation, calculus, abstraction.	from 0 to 30 Normal: ≥ 24 MCI: < 24
Questionnaire of functional activities from Pfeffer. Pfeffer et al.^10^	Ten questions about instrumental activities of daily living and cognitive-social functions. Answered by a companion (in person or by phone).	From 0 to 30 ≥ 5 = functional impairment
Mini/Mini-Plus** Lecrubier et al.^21^	Diagnostic interview based on the DSM-IV and ICD-10, subdivided into 12 steps with YES/NO answers, building an algorithm which result has diagnostic value for Depression.	See if you have something to write here or leave it like this: The result of the algorithm has diagnostic value for Depression.

CIC: cognitive impairment;

* Available in www.mocatest.org;

** May be used by clinicians, and copies are authorized for researchers and clinicians who work in universities, hospitals and governmental institutions.

### Magnetic resonance evaluation of the brain

We performed MRI images of the brain using a high-field Siemens/Avanto apparatus (1.5 Tesla), without paramagnetic contrast. For this purpose, we used a specific protocol recommended for the study of cognitive alterations and dementia^23^. We used three visual scales to assess the MRI images - Fazekas^24^, MTA (Mesial Temporal Atrophy)^25^ and the GCA (Global Cortical Atrophy)^26^ (Chart 2). The image analyses were performed by neuroradiologists blinded in the study.

**Chart 2:** Chart showing the scales used in MRI images

Scale/Score	0	1	2	3	4
FAZEKAS White matter lesion Leukoaraiosis/ microangiopathy Fazekas et al.^24^	No changes	Mild Scattered pointed foci	Moderate Initial confluence of foci	Severe Large, extensive and confluent areas	XXX
TMA Hippocampal atrophy Scheltens et al.^25^	No changes no atrophy	Only enlargement of the choroid fissure	Also, enlargement of the lateral ventricle lateral horn	Moderate loss of hippocampal volume (height reduction)	Severe loss of hippocampal volume
GCA Global cortical atrophy Pasquier et al.^26^	No changes no cortical atrophy	Mild sulci opening	Moderate Loss of gyri volume	Severe atrophy “knife blade”	XXX

TMA: temporal mesial atrophy; GCA: global cortical atrophy.

### Statistical analysis

We ran a descriptive analysis of the data with the mean, standard deviation, median or percentage according to the characteristic of the variable. We assessed normality using the Kolmogorov-Smirnoff test. A comparison was made between patients with normal MoCA vs. altered sociodemographic, clinical, laboratory, inflammatory and imaging variables, using the Chi-square and/or Student’s t tests for independent data, and the Mann-Whitney test to compare medians. The correlations between MoCA and eGFR with all variables were performed using the Pearson or Spearman correlation tests. Finally, we performed a linear regression using the MoCA as the dependent variable; and age, gender and eGFR as confounding variables. For each model, we used an inflammatory marker as an associated confounding variable, as there was a lot of collinearity between them, preventing them from being placed in a single model. We used the SPSS 17.0 software, Chicago, Illinois®.

## Results

**Figure 1:**
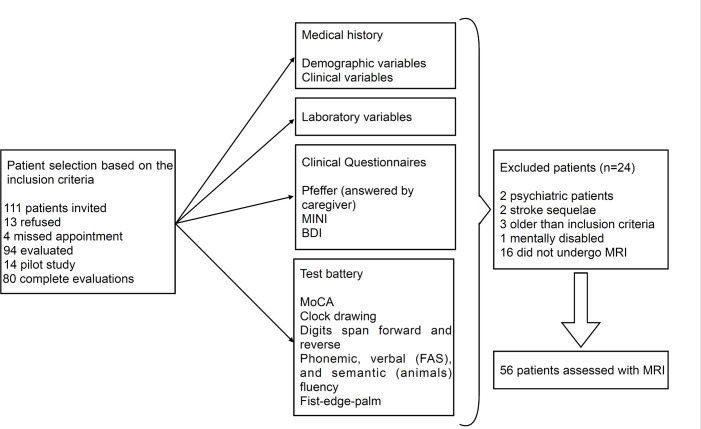
Study flowchart.

We invited 111 patients to participate in the study; 13 of whom refused, and four were scheduled but did not attend the evaluations. The remaining 94 signed the Consent Form and underwent the evaluations. Of these, 14 made up the pilot study, leaving eighty complete evaluations. After applying the inclusion criteria, eight patients were excluded and 16 refused to undergo brain MRI, making a final sample of 56 patients (Figure 1).

To present the results, the patients were divided into two groups: normal MoCA (≥ 24 points) and altered MoCA (< 24 points).

Regarding the sociodemographic parameters, there was a tendency to change the MoCA in older patients (58.2 ± 6.9 vs. 54.2 ± 9.2 years; p = 0.06), as well as in patients with schooling < 4 years, when compared to those > 4 years (p = 0.04). Regarding comorbidities, patients with DM tended to have an altered MoCA (65.5% vs. 38.5%, p = 0.06). In other comorbidities, as well as in the different CKD categories, there were no significant changes (Table 1).

**Table 1:** Comparison of sociodemographic and clinical variables in patients with altered vs. normal MoCA

Variable		MoCA altered	MoCA normal	p
	Age (mean; sd)	58.2 ± 6.9	54.2 ± 9.2	0.06
Sex (%)	Male	55.2	55.6	0.59
	Female	44.8	44.4	
Color (%)	White	41.4	50.0	0.68
	Black	24.1	15.4	
	Brown	34.5	34.6	
Marital status (%)	Married (stable union)	55.1	63.0	0.42
	Divorced/Separated	24.1	7.4	
	Single	6.9	14.8	
	Widow(er)	13.8	14.8	
Schooling	Mean; sd	52.2 ± 2.8	6.8 ± 2.8	0.04
	≥ 4 years (%)	82.8	96.2	0.12
	< 4 years (%)	17.2	3.8	
Income (MW) (%)	Up to 2	60.0	50.0	0.82
	2-4	33.3	38.9	
	> 4	6.7	11.1	
	BMI (%)	29.9 ± 4.7	31.2 ± 5.0	0.37
	MBP (mean; sd)	143.7 ± 25.1	147.1 ± 23.1	0.62
	DBP (mean; sd)	87.3 ± 13.6	91.9 ± 15.3	0.27
Comorbidities (%)	SAH	96.6	92.6	0.60
	DM	65.5	38.5	0.06
	CVD	36.0	39.1	0.52
	Depression (Mini Plus)	20.7	22.2	0.88
	Hypothyroidism	16.0	18.2	0.57
	Smoking	23.1	18.5	0.74
	Drinking	4.0	14.8	0.35
	Sedentarism	22.2	30.4	0.36
CKD category (%)	1	3.4	11.5	0.76
	2	6.9	7.7	
	3A	27.6	15.4	
	3B	34.5	42.3	
	4	24.1	19.2	
	5	3.4	3.8	
Medications being used (%)	Opiates	0	0	
	Anti-parkinsonians	0	3.8	0.43
	Hypnotics	3.6	0	0.52
	Antiepileptics	14.3	20.0	0.71
	Benzodiazepines	17.9	20.0	0.84
	Antidepressants	38.5	45.5	0.77
	ACEI	53.8	45.5	0.77
	ARB	65.5	46.2	0.12
	Betablockers	51.7	50.0	0.55
	Adren. blockers	10.7	19.2	0.31
	Vasodilators	3.4	3.8	0.72
	Calcium Chanel blockers	34.5	46.2	0.27
	Diuretics	92.3	68.2	0.04
	Statins	57.7	54.5	0.52
	Vitamin D	15.4	13.6	0.6
	Oral hypoglycemics	73.1	31.8	0.008
	Insulin	27.6	15.4	0.22
	Erythropoietin	3.8	0	0.54
	Iron	0	4.5	0.45
	Levothyroxine	7.7	9.1	0.86
	Nitrate	3.4	3.8	0.72
	ASA	75.9	34.6	0.002

SD (±): Standard Deviation; %: percentage; ≥: greater than or equal to; <: less then; MW: minimum wage; BMI: body mass index; SAP: systolic arterial pressure; DAP: diastolic arterial pressure; SAH: Systemic Arterial Hypertension; DM: diabetes mellitus; CVD: cardiovascular disease; CKD: chronic kidney disease; ACEI: angiotensin converting enzyme inhibitor; ARB: angiotensin receptor blockers; ASA: acetyl-salicylic acid.

As for the use of medications, there were significant changes in patients using diuretics (92.3 vs. 68.2; p = 0.04), oral antidiabetics (73.1 vs. 31.8; p = 0.008) and AAS (75.9 vs. 34.6; p = 0.002). There were no statistically significant values for the other medications (Table 1).

**Figure 2:**
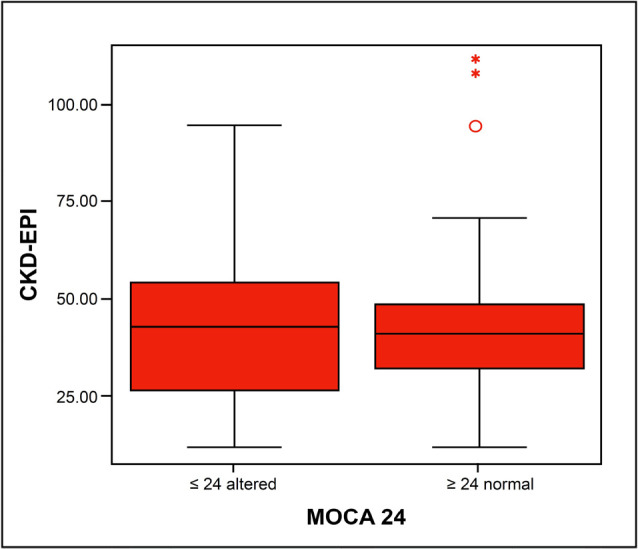
Graph of GFR by the formula CKDEPI vs. Altered and normal MoCA.

**Figure 3:**
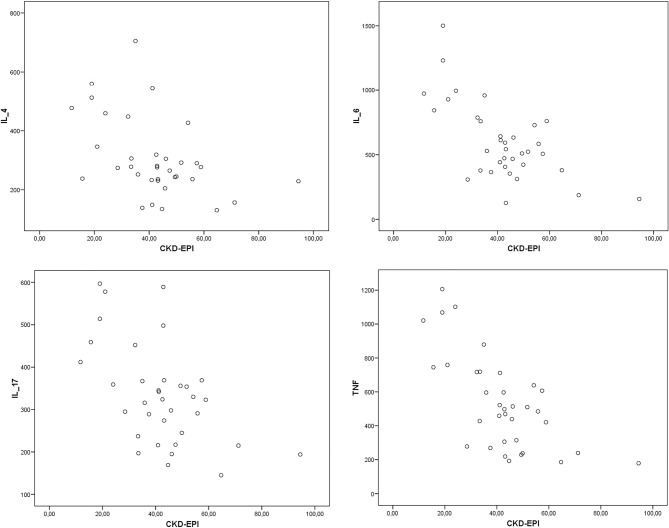
Scatter graphs showing correlations between eGFR by CKD-EPI and IL-4, IL-6, IL-17, TNF-α.

IL-4: interleukin 4; IL-6: interleukin 6; IL-17: interleukin 17; CKD-EPI: Chronic Kidney Disease Epidemiology Collaboration Equation.

In the laboratory evaluation, plasma sodium (138.0 vs. 139.6 mEq/L; p = 0.06) and ferritin (105.0 vs. 157.7; p = 0.06) were slightly higher in the group of patients with altered MoCA. However, these data were not statistically significant, or clinically relevant. The same was true in relation to triglycerides, which were lower in the group with altered MoCA (148.5 vs. 222.9 mg/dL; p = 0.06). Importantly, there was no statistically significant difference in the other exams, including eGFR (Table 2 and Figure 2). Furthermore, there were no associations between eGFR and the inflammatory variables (Figure 3).

**Table 2:** Comparison of laboratory variables in patients with altered MoCA compared to those with normal MoCA (cutoff point ≥ 24)

Variable		Altered MoCA mean/sd	Normal MoCA mean/sd	p
Creatinine	mg/dL	1.9 ± 1.0	2.1 ± 1.4	0.50
eGFR	mL/min/m^2^/SC	42.1 ± 18.9	45.8 ± 25.3	0.54
Proteinuria	g/24h	695.4 ± 1263.4	347.1 ± 316.6	-
Urea	mg/dL	58.1 ± 31.8	64.0 ± 37.9	0.59
Glucose	mg/dL	118.5 ± 47.1	122.7 ± 56.8	0.79
Hemoglobin Glycated	%	7.6 ± 2.8	8.2 ± 3.7	0.58
Sodium	mEq/L	138 ± 3.2	139.6 ± 2.7	0.06
Potassium	mEq/L	4.5 ± 0.7	4.7 ± 0.7	0.46
Magnesium	mg/dL	1.8 ± 0.3	1.7 ± 0.2	0.11
Uric acid	mg/dL	6.4 ± 1.4	6.9 ± 2.0	0.29
Calcium	mg/dL	9.6 ± 0.4	9.3 ± 0.8	0.23
Phosphorus	mg/dL	3.9 ± 0.8	3.6 ± 0.7	0.13
PTHi	pg/mL	153.7 ± 150.8	188.8 ± 243.9	0.57
Hemoglobin	g/dL	13.0 ± 1.9	13.5 ± 1.6	0.47
Ferritin	μmol/L	105.0 ± 83.3	157.7 ± 95.7	0.06
TSI	%	26.9 ± 10.6	29.9 ± 8.8	0.33
ALT	UI/L	22.0 ± 22.3	16.7 ± 9.9	0.38
AST	UI/L	28.0 ± 21.1	21.6 ± 7.2	0.26
Total cholesterol total	mg/dL	158.8 ± 43.5	171.4 ± 37	0.32
HDL	mg/dL	41.5 ± 10.8	37.0 ± 8.2	0.13
LDL	mg/dL	126.4 ± 54.3	116.3 ± 45.9	0.54
Triglycerides	mg/dL	148.5 ± 84.1	222.9 ± 148.9	0.05
Albumin	g/dL	4.0 ± 0.38	4.0 ± 0.4	0.62
TSH	mUI/L	3.7 ± 4.4	2.2 ± 1.9	0.16
Vitamin B12	pg/mL	363.5 ± 171.7	351.14 ± 155.5	0.81
Folic acid	ng/mL	9.3 ± 3.6	8.7 ± 3.5	0.60
Vitamin D3	ng/mL	31.0 ± 6.6	27.9 ± 7.5	0.23
VDRL	pos./neg.	4.8	0	0.24
FTA-ABS	pos./neg.	9.5	0	0.10

t-test for independent samples or Chi-squared; (±): standard deviation; mg/dL: milligrams per deciliter; eGFR: estimated glomerular filtration rate; mL/min/m^2^ SC: milliliter per minute per square meter; g/24h: grams in 24 hours; %: percentage; mEq/L: milliequivalent per litter; PTHi: intact parathormone; pg/mL: picogram per milliliter; g/dL: gram per deciliter; μmol/L: micromole per liter TSI: transferrin saturation index; IU/L: International units per litter; ALT: alanine aminotransferase; AST: aspartate aminotransferase; HDL: high density lipoprotein; LDL: low density lipoprotein; TSH: thyroid stimulating hormone; mUI/L: international milliunits per litter; ng/mL: nanogram per milliliter; VDRL: Venereal Disease Research Laboratory; FTA-ABS: fluorescent treponeme antibodies.

Neuroimaging was performed by MRI, the gold standard for evaluating the white matter lesions by detecting microangiopathies or leukoaraiosis, MCI markers of a vascular nature. These lesions, evaluated by the Fazekas scale,^24^ had a score of 0 in 17.1% of the cases; and a score of 1 in 60.7%, that is, mild or no changes in the white matter, not explaining a CI of a vascular nature. Moderate microangiopathy or Fazekas 2 were found in only 19.6% of the cases. However, as they are mostly hypertensive patients, SAH per se would justify the presence of these alterations.

There were no changes (MTA 0) in the MTA^25^ scale, used to assess mesial changes in the temporal lobes, correlated with Alzheimer’s disease, in 81.8% of the cases, and 16.4% had mild changes. Thus, similarly to the previous scale, there were no alterations related to Alzheimer’s disease detected using the MTA scale.

The same behavior was observed in the third scale used, the ACG^26^. In 98.2% of the cases, cortical atrophy ranged between 0 (74.5%) and 1 (23.6%), that is, there was no atrophy in most cases and, when present, it was mild. Only 1.8% had severe cortical atrophy. Therefore, there were no statistically significant image changes in the normal MoCA and altered MoCA groups (Table 3).

**Table 3:** Comparison of the Fazekas, mta and acg scales in patients with altered vs. normal MoCA (cutoff point ≥ 24) per chi-square

Data		Altered MoCA	Normal MoCA	p
FAZEKAS (%)	0	17.2	18.5	0.67
	1	62.1	59.3	
	2	20.7	18.5	
	3	0	0	
	Others	0	3.7	
MTA (%)	0	72.4	92.3	0.11
	1	24.1	7.7	
	2	3.4	0	
	3	0	0	
	4	0	0	
GCA (%)	0	65.5	84.6	0.18
	1	31.0	15.4	
	2	0	0	
	3	3.4	0	
OTHER FINDINGS (%)	Ischemic gap	20.7	11.5	0.72
	Micro-hemorrhages	10.3	7.7	
	Other	13.8	11.5	

≥: greater than or equal to; %: percentage; MTA: Mesial Temporal Atrophy; GCA: Global Cortical Atrophy.

**Figure 4:**
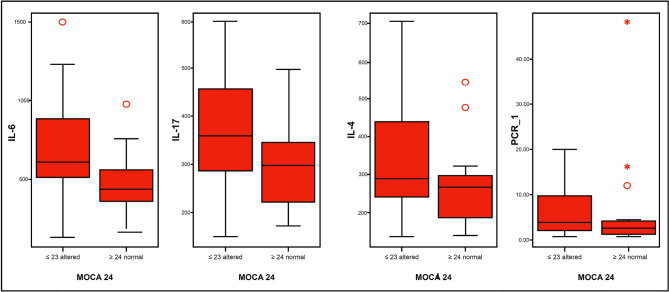
Altered MoCA vs. normal and variable means inflammatory.

IL-4: interleukin 4; IL-6: interleukin 6; IL-17: interleukin 17.

Table 4 and Figure 4, show important differences between the levels of IL-6, IL-17 and TNF-α between patients with altered MoCA, compared to normal.

**Table 4:** Comparison among laboratory inflammatory variables in patients with normal vs. altered MoCA (cutoff point ≥ 24) per test for independent samples

Data	Altered MoCA	Normal MoCA	p
IL-4	328.7 ± 116.4	283.0 ± 88.9	0.10
IL-6	666.0 ± 277.2	529.2 ± 160.0	0.02
IL-17	360.0 ± 109.2	311.0 ± 70.3	0.05
TNF-α	588.3 ± 248.8	479.3 ± 180.6	0.06
CRP-us	7.0 ± 5.2	7.7 ± 9.3	0.73

IL-4: interleukin 4; IL-6: interleukin 6; IL-17: interleukin 17; TNF-α: tumor necrosis factor alpha; CRP-us: ultrasensitive C-Reactive Protein.

In the linear regression analysis models (Table 5) using the MoCA as the dependent and continuous numerical variable, all models were adjusted for age, sex and eGFR. The variables IL-4, IL-6, IL-17 and TNF-α were not placed in the same model because of their high collinearity; therefore, each model was adjusted for each of these variables separately. The model was not adjusted for the DM variable, because this is a population that has a different behavior.

**Table 5:** Linear regression (MoCA-dependent variable) adjusted for age, sex, eGFR and in model 1 for IL-17, model 2 IL-4, model 3 IL-6 and model 4 TNF alpha (n=56)

Models	Variable	B	p	Lower CI	Higher CI
Model 1	Age	0.44	0.608	-0.131	0.219
	Male	2.99	0.037	0.191	5.806
	eGFR	-0.083	0.082	-0.178	0.011
	IL-17	-0.018	0.026	-0.034	-0.002
Model 2	Age	-0.010	0.911	-0.189	0.169
	Male	2.360	0.113	-0.604	5.324
	eGFR	-0.0034	0.424	-0.120	0.052
	IL-4	-0.009	0.226	-0.024	0.006
Model 3	Age	0.001	0.989	-0.163	0.165
	Male	2.450	0.076	-0.280	5.180
	eGFR	-0.064	0.127	-0.147	0.020
	IL-6	-0.006	0.023	-0.012	-0.001
Model 4	Age	0.010	0.913	-0.176	0.196
	Male	2.470	0.098	-0.495	5.434
	eGFR	-0.050	0.308	-0.149	0.049
	TNF-α	-0.005	0.200	-0.012	0.003

eGFR: estimated glomerular filtration rate; IL-17: interleukin 17; IL-4: interleukin 4; IL-6: interleukin 6; TNF-α: tumor necrosis factor alpha.

In model 1, adjusted for IL-17, males and IL-17 were independently associated with MoCA, with males being positively associated, and IL-17 being negative. In model 2, adjusted for IL-4, there was no association with MoCA. Model 3, adjusted for IL-6, showed that only this inflammatory marker was an independent predictor of altered MoCA. The last model, adjusted for TNF-α, did not show any association.

## Discussion

The hypothesis that cognitive alterations in CKD are initially of a functional nature and, therefore, not identifiable by MRI was observed in our study, also the CI assessed by MoCA was prevalent in patients with pre-dialysis CKD and associated with inflammatory markers, the same not occurring in relation to MRI findings.

The sample consisted of patients with a lower mean age (56.3 ± 8.3 years) than that observed in the usual population of the pre-dialysis CKD outpatient clinic (66.2 ± 13.4 years) of the CHM-JF^27^ In this study, the population presented, as a non-inclusion criterion, age > 65 years, considering that the increased incidence and prevalence of vascular and degenerative diseases occur from this age group onwards. This approach substantially reduced the age bias in the study sample. On the other hand, we know the action of diseases reported to cause vascular aging and early atherosclerosis in these individuals^18,28^.

Regarding education, the MoCA was lower in those with < 4 years of education when compared to those > 4 years. The sample compared to the National Household Sample Survey (PNAD), 2019, revealed a higher percentage of individuals with more than 4 years of education (89.1% vs 32.2%)^29^. There is evidence in the literature that correlates MoCA with education, being significantly higher in individuals with high education, thus resulting in the cutoff point of 26 used in the original study, contrasting with other studies validated in other countries that used various cut-off points. The higher the educational level, the greater the cognitive reserve and the better the performance on tests, thus acting as a protective factor for dementia^30^. In this study, we adopted the cutoff point of 24, as it has already been suggested in previous studies in the Brazilian population^22,31^ and in patients with CKD^32^.

As for comorbidities related to CKD, there were significant changes found in patients with DM, a population known to be at higher vascular risk. A study by Freedman et al.^33^ using secondary data evaluated the relationship between cognition and DM with MRI findings, and reported a worse performance on the MMSE associated with a decrease in gray matter, with a higher albumin/creatinine ratio and a reduction in eGFR. Our study found no association of proteinuria or eGFR with CC assessed by MoCA.

Due to the high prevalence of SAH in our sample, we could not establish its association with CI, although there are numerous citations that correlate SAH as one of the main traditional risk factors for vascular compromise and secondarily to CI and CKD itself^18,19,34^.

Regarding the use of medications, there were significant changes in patients using oral antidiabetics, which, as discussed above, cause worse cognitive performance. Another therapeutic class associated with worse cognitive performance was diuretics. This fact may be related to the greater use of this therapeutic class in patients in more advanced categories of CKD and the likely association of CKD with other comorbidities. A systematic review^34^ addressing the use of antihypertensive drugs and cognition in the elderly, despite highlighting the heterogeneity of the studies, showed an association between the use of ARB and improvements in episodic memory, while diuretics and other antihypertensive drugs did not have any association^34^. The same occurred with the use of acetylsalicylic acid, associated with a higher prevalence of CVD and, for this reason, it is believed to be associated with worse MoCA performance.

As for laboratory findings, the relationship between CI and anemia in CKD is a known fact. The drop in ferritin is related to worse functionality, lower MoCA scores in verbal and working memory, and in attention^35^. In this study, higher ferritin values were associated with better MoCA performance, but it seems that this increase is not associated with inflammation, but with iron reserves. In our study, we can speculate that hypertriglyceridemia may be associated with CKD, a condition in which dyslipidemia is observed in up to 75.7% of cases, and hypertriglyceridemia in up to 50%^36^.

Previous studies^20,37-38^ have shown a correlation between impaired renal function and CI, but most of these studies were carried out in populations over 65 years of age, in whom CKD is associated with the aging process. In the literature, it has been reported that CI, including dementia, occurs in 16% to 38% of patients with end-stage CKD^38^. In the REGARDS study^37^, a cohort with 23,405 patients, with a mean of 64.9 years, they reported that, in cases of eGFR < 60 mL/minute, 10 mL reduction in eGFR increased by 11% the prevalence of cognitive decline. Another prospective cohort^38^ showed a correlation between worsening renal function and CI in a multibreed population, with eGFR less than 90 mL/minute and follow-up for 2.8 years; and they concluded that CKD was linearly associated with cognitive decline in this population, adjusted for multiple risk factors, even in patients with slightly reduced renal function^38^. On the other hand, the CRIC^39^ study in younger patients with mild to moderate CKD (57.7 ± 11.0 years), with a mean eGFR of 45.0 ± 16.9 mL and a mean of 6 years of follow-up, concluded that CI is not associated with additional risk of CKD progression. In our study, consisting of patients under 65 years of age and with a predominance of earlier CKD categories, there was an important prevalence of CI, but this was not linearly associated with eGFR.

A relevant finding was the association of inflammatory markers with MoCA, especially in linear regression, with significant associations of dosages in the increase of pro-inflammatory cytokines IL-6 and IL-17, as well as a statistical trend in TNF-α. The same was did not happen for the anti-inflammatory cytokine IL-4. These findings corroborate those in the literature that report the importance of inflammation in the context of CKD, and the likely involvement of the endothelium and, secondarily, of the blood-brain barrier (BBB)^19,28^, initially leading to functional changes and, subsequently, to the worsening of CKD and structural vascular lesions. A systematic review^40^ on the association between IL-6 and CKD concluded that IL-6 is directly related to the progression of CKD, which may be a possible marker of kidney damage to be used both in the diagnosis and in the follow-up of this population. Similarly, it can be speculated that, based on the data from the present study, IL-6 may also be a marker of CI in CKD.

Numerous publications^16,18,19,24,28,33^ correlate CI in CKD with vascular lesions, especially those located in the white matter, secondary to compromised microcirculation (microangiopathic or small vessel vascular disease) resulting from this fact, the use of neuroimaging as a diagnostic resource, with emphasis on MRI, which, when compared to CT, has greater sensitivity in the early detection of these lesions, considered specific markers of CI of a vascular nature. Studies correlating white matter lesions or microangiopathies (leukoaraiosis) associated or not with microinfarcts, microbleeds and cortical atrophies with CI^16,18,19,28,33^ are frequent. Differently in the present study, there was no correlation between altered MoCA and MRI findings, as the images were within normal limits or with very subtle alterations, not justifying the CI detected in 51.8% of the patients evaluated. This discrepancy could be explained by the functional nature of these alterations that precede the anatomical ones reported in previous studies. On the other hand, when CI was compared to inflammatory markers, a direct relationship was found with pro-inflammatory cytokines, especially IL-6. These findings suggest the hypothesis of the functional nature of CI in the population evaluated.

Among the limitations of the present study is the fact that this is a cross-sectional study, carried out in a single center and with a small sample size. On the other hand, it is worth mentioning as positive points the performance of MRI in all patients, in addition to the dosage of inflammatory markers.

A fact of clinical relevance was the demonstration of the MoCA usefulness, a simple, easy-to-perform and low-cost tool that can be applied by non-specialized professionals^9,31^. The addition of this tool to the clinical routine of nephrologists may contribute to the early diagnosis of CI and enable the implementation of preventive and therapeutic measures in patients with chronic kidney disease.

## Conclusion

The MCI assessed by MoCA was prevalent in patients with pre-dialysis CKD, was associated with inflammatory markers and did not show any correlation with changes seen in the MRI.
